# Determining Skin Cancer Protective Behaviors in the Light of the Protection Motivation Theory among Sailors in Bandar-Bushehr in the South of Iran

**DOI:** 10.31557/APJCP.2020.21.12.3551

**Published:** 2020-12

**Authors:** Ahmad Sotoudeh, Seyed Saeed Mazloomy Mahmoodabad, Ali Akbar Vaezi, Mojtaba Fattahi Ardakani, Reza Sadeghi

**Affiliations:** 1 *Department of Public Health, Bushehr University of Medical Sciences, Bushehr, Iran. *; 2 *Department of Health Education and Promotion, Social Determinants of Health Research Center, School of Public Health, Shahid Sadoughi University of Medical Sciences, Yazd, Iran. *; 3 *Department of Nursing, School of of Nursing & AMP, Midwifery, Research Center for Nursing & AMP, Midwifery Care in Family Health, Shahid Sadoughi University of Medical Science, yazd, Iran. *; 4 *Yazd Diabetes Research Center, Shahid Sadoughi University of Medical Sciences, Yazd, Iran. *; 5 *Department of Public Health, Sirjan School of Medical Sciences, Sirjan, Iran. *

**Keywords:** Skin cancer, sailors, risk reduction behavior, protection motivation theory

## Abstract

**Background:**

Skin cancer is among the most prevalent cancers in Iran and worldwide. Due to the nature of work, sailors are constantly exposed to the ultraviolet rays of the sun, which in the long run damages their skin and raises the chances of skin cancer. Thus, the present research aimed to predict the skin cancer protective behaviors among sailors in the south of Iran in the light of the protection motivation theory.

**Materials and Methods:**

The present analytical, cross-sectional research was conducted on 360 sailors in Bandar-Bushehr selected randomly from 4 border healthcare centers. To collect the required data, a reliable and valid questionnaire based on the protection motivation theory was used. The data were analyzed in SPSS21 using descriptive and inferential statistics including Pearson correlation coefficient and linear regression analysis.

**Results:**

Pearson correlation coefficient showed a statistically significant positive correlation between protection motivation, perceived severity, fear, reward on the one hand and a statistically significant negative correlation between protection motivation and rewards and response costs. All constructs of protection motivation explained 43% of the variance of skin cancer protective behaviors. Among the influential predictors, perceived self-efficacy showed to be the strongest (β=0.328).

**Conclusion:**

Considering the effectiveness of the protection motivation theory in determining skin cancer preventive behaviors among sailors, it can be concluded that this theory can be used as a framework in planning health education and promotion programs to motivate sailors to adopt more skin cancer protective behaviors.

## Introduction

Skin cancer is the most prevalent type of cancer worldwide and accounts for at least 40% of all cases of cancer (Cakir et al., 2012). About 5 million people annually receive medical care in the Unites States for this disease (Lomas et al., 2012). Skin cancer is generally divided into two types: melanoma and nonmelanoma skin cancer. The latter non melanoma skin cancer (NMSC) is known as the most prevalent type in the world (Organization, 2014, Lomas et al., 2012). A body of research has shown that the occurrence rate of the disease is ever increasing in Iran, especially among outdoor workers, who are more exposed to the sunlight and more particularly the ultraviolet ray (Fartasch et al., 2012, Szewczyk et al., 2016). As the related literature showed, the foremost environmental risk factor of all skin cancers is the ultraviolet radiation, either originating from the sun or any artificial source (Day et al., 2013). Sailors are among the primary populations at risk as they are required to work for long hours during the day in sunlight. If they are not equipped with appropriate covering and shields to protect them against sunlight, they are highly prone to skin cancer (Grandahl et al., 2018, Sotoudeh et al., 2019). 

There is a dearth of research on skin cancer preventive behaviors among outdoor workers in Iran. Some research on Iranian farmers showed a statistically significant correlation between all constructs of the protection motivation theory and skin cancer preventive behaviors (Babazadeh et al., 2017). Thus, it is essential to take sailors’ perceptions into account in planning and developing educational programs. Sun protective behaviors are essential to prevent skin cancer (Baghianimoghadam et al., 2012). 

It is noteworthy that health education theories and models provide a conceptual framework for the correlates of a health issue on a regular and logical basis (Tsai et al., 2016). The protection motivation theory (PMT) is primarily used to appraise cancer preventive perceptions and behaviors (Conner and Norman, 2005). PMT is suited for adopting skin cancer preventive behaviors, as it has a motivational component. This theory was introduced to explain determining protective behavior and can be used to change attitude and behavior. As assumed in this theory, to show a particular behavior, people consider the threats and benefits. Thus two processes are involved, threat appraisal and conflict appraisal to see whether people go for protective behaviors or high-risk behaviors. The first one is an initial cognitive process which involves appraising perceived severity and vulnerability to current threat and rewards for current activities, and may inhibit risk protective behaviors. Perceived severity and vulnerability deal more with the promotion of behavior, while perceived external and internal rewards tend more to prevent the protective behavior. The second cognitive process (i.e. conflict appraisal) involves appraising perceived self-efficacy, perceived response efficacy and perceived costs of the preventive act. The higher perceived self-efficacy and response efficacy and the lower the perceived costs, most likely promote the higher chances of protective behavior against sunlight (Rippetoe and Rogers, 1987, Sadeghi et al., 2019). 

Further researches are needed to demonstrate sunlight protective strategies among sailors. Thus, the present research aimed to explore skin cancer protective behaviors based on the protection motivation theory among sailors in Bandar-Bushehr. The resent findings can shed light on potential opportunities and capabilities of promoting health education and skin cancer preventive interventions among sailors and coastal residents.

## Materials and Methods

*Research design and sampling*

The present descriptive cross-sectional research was conducted on 362 sailors having health records in border healthcare centers in Bushehr province in the south of Iran in 2019. The sampling was simple randomized in type. Thus, 2 healthcare centers were randomly selected from 7 border healthcare centers. The research participants were finally selected randomly from among the existing health records in the healthcare center. The sample size was set guided by the related literature (Babazadeh et al., 2017).(SD=6.5, SEM=.7, CI=95%) via the following formula: n=z^2^s^2^/d^2^. The estimated sample size was 350, yet for a better fitness, it was decided to be increased to 370. Eight participants were excluded as they did not complete the questionnaire. Eventually, the data collected from 362 participants were statistically analyzed. The data collection procedure was done between October 5, 2019 and November 28, 2019. 

*Instrumentation*

The data collection instrument was a researcher-made questionnaire developed in the light of the related qualitative research (Mahmoodabad et al., 2019, Sotoudeh et al., 2019, Zink et al., 2018) There were 3 parts to the questionnaire, the first of which enquired about respondents’ demographic information (age, education level, marital status, family size, income, insurance coverage, sea work experience, history of sunburn, history of skin cancer among relatives). The second part dealt with the constructs of PMT in preventing skin cancer. These constructs included perceived susceptibility (5 items, R=5-25), perceived severity (5 items, R=5-25), perceived self-efficacy (6 items, R=6-30), perceived response cost (5 items, R=5-25), response efficacy (6 items, R=6-30), perceived rewards (4 items, items, R=4-20), fear (4 items, R=4-20) and protection motivation (7 items, R=7-35) all rated on a 5-level Likert scale ranging from 1 (strongly disagree) to 5 (strongly agree). The third part comprised 9 items exploring skin cancer preventive behaviors such as information acquisition, hours of exposure to sunlight, use of such protections as sunscreen, gloves, cap, long sleeves, sunglasses and duration of wearing sunscreen. These items were responded to as Yes or No. A Yes would receive 1 and No would receive 0. Thus, the range of scores was 0 to 9 and a higher score showed a higher rate of the skin cancer preventive behavior. For content validity, a panel of experts was consulted. As concluded from experts’ comments, content validity, simplicity and unambiguity/clarity were rated as 88%, 93% and 91%, respectively, all being acceptable. In order to test the reliability of test scores, the questionnaire was submitted to a sample of 30 sailors and the Cronbach’s alpha test of internal consistency was run. The estimated value was .86, .83, .86, .93, .81, .88, .79 and .83 for perceived susceptibility, severity, self-efficacy, rewards, response cost, fear and protection motivation, respectively, all being acceptable (P<0.005).

*Statistical analysis*

The data were analyzed in SPSS21. Descriptive statistics were used besides Kolmogorov-Smirnov‘s test of normality. It was followed by ANOVA and Independent-samples T-test to see how sailors’ practice diverged across demographic variables. Moreover, Pearson’s correlation coefficient was used to test the correlation between the constructs of PMT and skin cancer preventive behaviors. Linear regression analysis was used to determine the key predictors of protection motivation among sailors. The significance level was set at 0<0.05.

*Ethical considerations*

To abide by ethical rules, participation in this research was quite voluntary and via fully informed consent. All throughout the procedures, the participants were ensured of the confidentiality of the information they provided. The questionnaire completion was anonymous. The research protocol was approved by the committee of ethics at Shahid Sadoughi University of medical sciences in Yazd (IR.SSU.SPH.REC 1397.085).

**Table 1:** Scio Demographic Characteristics of the Respondents (n=362)

Characteristics		N	%
Age	Less than 30 years	112	30.9
	From 30 to 40	113	31.2
	From 41 to 50	66	21.3
	More than 51 years	60	10.6
Educational Level	Primary	94	26
	High school	121	33.4
	Diploma	126	34.8
	Collegiate	21	5.8
Marital Status	Single	83	22.9
	Married	270	74.6
	Widowed	9	2.4
Income Status	Good	25	6.9
	Average	151	41.7
	Weak	186	51.4
Insurance Services	Rural	114	31.5
	Social Security	185	51.1
	Medical	7	1.9
	No Insurance	56	15.5
Medical Insurance Services	Yes	13	3.6
	No	349	96.4
Work Experience	Less than 5years	139	38.4
	From 5 to 10	135	37.3
	More than 10 years	88	24.3
History of Sunburn	Yes	192	53.0
	No	170	47.0
History of Skin Cancer among Relatives	Yes	11	3.0
	No	351	97

**Table 2:** Correlation of PMT Constructs in the Descriptive Research

PMT constructs	Perceived susceptibility	Perceived severity	Fear	Self efficacy	Response efficacy	Response cost	Rewards	Protection motivation
Perceived susceptibility	1	**						
Perceived severity	0.328**	1						
Fear	0.512	0.190*	1					
Selfefficacy	0.421	0.390**	-0.185*	1				
Response efficacy	0.101	0.180*	-0.0189*	0.658**	1			
Response cost	0.036	0.302**	-0.094	0.538**	0.162	1		
Rewards	-0.079	0.514**	0.158	0.034	0.189*	-0.203**	1	
Protection motivation	0.034	0.179*	0.248**	-0.094	-0.096	-0.080	0.248	1

*Correlation is significant at the 0.05 level (twotailed), **Correlation is significant at the 0.01 level (twotailed); PMT, Protection motivation theory

**Table 3:** Multivariate Linear Regression Analysis of Effective Demographic Factors in Protective Behaviors

Variable	β	SE (β)	Standardized β	t	P	Lower	Upper
Final model							
Age	0.42	0.06	0.301	3.78	0.004	0.11	0.39
Level of education	0.856	0.319	0.125	3.21	0.001	-0.356	1.592
Work experience	0.514	0.248	0.183	2.127	0.041	0.026	0.878
History of sunburn	0.646	0.235	0.265	2.87	0.003	0.25	1.23

**Table 4:** Multiple Linear Regression Analysis of Protection Motivation Theory Constructs Effective on Skin Cancer Protective Behaviors

Variable	R^2^	B	SE	BETA	P
Protection motivation	43%				
Perceived susceptibility		0.002	0.032	0.007	0.805
Perceived severity		0.035	0.025	0.087	0.148
Rewards		-0.002	0.012	-0.021	0.624
Response efficacy		0.081	0.028	0.161	0.014
Selfefficacy		0.435	0.06	0.328	0
Response cost		-0.058	0.017	-0.118	0.068

## Results

The total number of participants in this research was 362 and their mean age was 37.96±10.44 years. From among this number, 112 (30.9%) 

Were below 30 years of age, 113 (31.2%) between 30-40 years, 77 (21.3%) between 41 and 50 years and 60 (16.6%) were above 50 years old. As for education majority 34.8% high school degree and majority of participants 97% had no history of skin cancer among relatives Table 1. 

Pearson’s correlation coefficient showed a statistically significant correlation between perceived susceptibility, severity and fear. Similarly, a significant positive correlation was found between fear and perceived severity. Moreover, perceived self-efficacy and severity showed to be positively correlated. Perceived response efficacy is positively correlated with perceived severity and self-efficacy. Perceived response cost, perceived severity and self-efficacy were positively correlated. The analyses also revealed that perceived rewards, severity and response efficacy were positively correlated. Furthermore, protection motivation showed to be positively correlated with perceived severity, fear and rewards.

The analyses revealed that perceived self-efficacy and fear were negatively correlated. Similarly, perceived response efficacy, susceptibility and fear showed to be negatively correlated. The results further showed a statistically significant negative correlation between perceived rewards and response cost Table 2. 

Regression analysis of factors influencing protective behaviors showed a statistically significant correlation between age and protective behaviors (β=.301, p=.001). The mean behavior scores were the lowest in the >50 age group among all. A statistically significant correlation was found between education level and protective behaviors (β=0.125, p=0.001). The mean protective behavior score was the highest among those with a high school degree. A statistically significant correlation was found between work experience and protective behaviors (β=0.183, p=0.041). In fact, in the 5-10 year work experience group, the mean score of protective behaviors was significantly higher than those below 5 years of experience or above 15 years. Moreover, a statistically significant correlation was found between history of sunburn and protective behaviors (β=0.235, p=0.003). The mean score of protective behaviors was significantly higher among those with a history of sunburn Table 3.

Linear regression analysis showed perceived self-efficacy and response efficacy as the significant predictors of protection motivation. This regression model managed to explain 43% of variation in protection motivation. From among the effective factors, perceived self-efficacy was found to be the strongest predictor (β=0.328) Table 4.

## Discussions

The present research aimed to determine skin cancer protective behaviors among sailors in the light of the protection motivation theory in Bandar-Bushehr, Iran. A sound knowledge of protective behaviors and recognition of effective factors contribute to the development of interventions to improve protective behaviors. As the present findings revealed, perceived barriers and challenges of protective behaviors (perceived response cost) and neglect of protective behaviors (perceived rewards) can significantly reduce the rate of skin cancer protective behaviors. On the other hand, sailors’ belief in the chances of affliction with skin cancer (perceived susceptibility), belief in their own capabilities of showing protective behaviors (perceived self-efficacy) and their perception of effective protective behaviors (perceived response efficacy) help to increase the rate of protective behaviors. Regression test results showed that sailors’ belief in their own capabilities of adopting protective behaviors and their perception of the effectiveness of protective behaviors are the only predictors of skin cancer preventive behaviors. 

In the present research, a significant positive correlation was found between perceived susceptibility, severity and fear. These findings were consistent with a body of research by Afshari et al., (2016), Kok et al., (2010) and Moeini et al., (2019). The research findings by Park et al., (2010) were not consistent with the present research. This divergence is probably due to the different research populations involved. That is to say that to increase sailors’ perceived threat of skin cancer, there is probably a need for increasing skin cancer susceptibility, which in turn increases the perceived threat of skin cancer. Moreover, as the results showed, a significant positive correlation was found between self-efficacy and perceived severity. To explain this correlation, it can be acknowledged that the more trust people put in their own capabilities of adopting protective behaviors, the better their perceived severity of the disease and the higher the chances of adaptive behaviors. These findings were consistent with those of several studies (Baghianimoghadam et al., 2012, Moradhaseli et al., 2019, Searle et al., 2000). In other words, sailors who perceived themselves self-efficacious in adopting protective behaviors, tended to show these behaviors more.

The present research revealed a significant positive correlation between perceived response efficacy, self-efficacy and severity. In fact, a higher response efficacy score is followed by a higher rate of perceived self-efficacy and severity. That is to say that the more one believes that an adaptive response can remove threat, the higher the chances of adopting protective behaviors. These findings are consistent with a body of research by Mohammadi et al., (2010), Moradhaseli et al., (2019) and Tazval et al., (2016). Yet, they were inconsistent with some research by Dasgupta et al., (2012). If sailors perceive the effectiveness of sunlight protective behaviors well, they go for protective behaviors more.

In the present research, protection motivation showed to be positively correlated with perceived severity and rewards. These findings were consistent with a body of research by Afshari et al., (2016), Baghianimoghadam et al., (2012) and Sadeghi et al., (2019). Yet, these findings were inconsistent with the results reported by Morovati Sharifabad et al., (2009). This divergence might be due to different research populations. Thus, the better one’s attitude toward protection motivation, the more motivated one becomes to adopt protective behaviors. This is only made possible when one’s perception of the social and physical side effects of skin cancer is increased and a positive sense of fear is produced. In the present research, perceived costs showed to be significantly and negatively correlated with perceived rewards and protection motivation. In other words, the higher the sailors’ perceived barriers, challenges and costs of protective behaviors, the higher the rewards and the lower the protection motivation. This is consistent with the results reported by Moeini et al ., (2019) and Afshari et al., (2016) Yet, the finding was not in agreement with Khorsandi et al., (2016). Higher perceived response cost is considered as a barrier to protective behaviors. Thus, a recognition of barriers, response costs and cutting down on them help to increase adaptive behaviors among sailors.

Moreover, according to the present findings, from among the demographic factors, age, education level, work experience and history of sunburn showed to be significantly correlated with the adoption of protective behaviors. Similarly Lowe et al., (2000), Morowatisharifabad et al., (2015), Moeini et al., (2019) and Tazval et al., (2016) reported that higher age is accompanied by less protective behaviors. It can be inferred that the elderly especially among sailors do not attend much to appearance and thus adopt less protective behaviors. Another finding of the present research was the statistically significant correlation between protective behaviors and sailors’ education level. Those with a high school degree acquired a higher mean score of protective behaviors than lower degrees. Similarly Morowatisharifabad et al., (2015) and Tazval et al., (2016) reported similar findings. As the results showed, higher education was accompanied by a higher and better perception of protective behaviors and their effect on the prevention of skin cancer.

In the present research, those with a history of sunburn had a higher mean score of protective behaviors. This finding was in line with what Moeini et al., (2019) found and reported. So were the results reported by Khorsandi, (2016), Moradhaseli et al., (2019). This would pinpoint that sailors with a history of sunburn adopt more protective behaviors on later occasions when exposed to the ultraviolet radiation. 

The present findings showed that the two constructs, perceived self-efficacy and perceived efficacy were the strongest predictors of skin cancer protective behaviors. These two constructs managed to explain 43% of variability in sailors’ protection motivation. Perceived self-efficacy was found as the strongest predictor of protection motivation. In a similar fashionstated self-efficacy as the best predictor of behavioral intention Hodgkins and Orbell, 1998, Tazval et al., 2016). These findings revealed that self-efficacy should be applied in the design of educational interventions to increase individuals’ capabilities.

In conclusion, in the light of the present findings it can be concluded that preventing skin cancer among sailors is essential due to their critical work conditions. Preventive measures and interventions should help recognize and implement factors affecting sailors’ adoption of skin cancer preventive behaviors. Besides, healthcare providers should receive educational programs to prevent skin cancer. As perceived self-efficacy and response efficiency were among preventive variables of skin cancer among sailors, it is suggested that the benefits of adopting protective behaviors be reminded such as less skin diseases, sunburn and skin cancer. Unfortunately, such cases as safe work environment and regular use of protections are lacking among sailors during their work hours in harbor and in the whole health system. Thus, these programs seem to be essential in the routine work plans of relevant organizations.

One limitation of the present research was the self-reporting nature of data. Moreover, this research was conducted on a specific research population (sailors) and the results might be incomparable to other population’s especially outdoor workers. A strength of the present research is the high participation rate of sailors as the participation was 91%. 
